# 24-hour human urine and serum profiles of bisphenol A following ingestion in soup: Individual pharmacokinetic data and emographics

**DOI:** 10.1016/j.dib.2015.03.002

**Published:** 2015-03-17

**Authors:** Justin G. Teeguarden, Nathan C. Twaddle, Mona I. Churchwell, Xiaoxia Yang, Jeffrey W. Fisher, Liesel M. Seryak, Daniel R. Doerge

**Affiliations:** a Health Effects and Exposure Science, Pacific Northwest National Laboratory, Richland, WA 99352, USA; b Department of Environmental and Molecular Toxicology, Oregon State University, Corvallis, OR 93771, USA; c Division of Biochemical Toxicology, National Center for Toxicological Research, U.S. Food and Drug Administration, Jefferson, AR 72079, USA; d Division of Epidemiology, College of Public Health, The Ohio State University, Columbus, OH 43210, USA

## Abstract

Here we present data to evaluate potential absorption of Bisphenol A through non-metabolizing tissues of the upper digestive tract. Concurrent serum and urine concentrations of d6-BPA, and its glucuronide and sulfate conjugates, were measured over a 24 h period in 10 adult male volunteers following ingestion of 30 μg d6-BPA/kg body weight in soup. The pharmacokinetic behavior of BPA and its metabolites in this cohort (rapid absorption, complete elimination, evidence against sublingual absorption) was reported. This Data in Brief article contains the corresponding individual pharmacokinetic data, reports the demographics of the cohort and provides additional details related to the analytical methods employed and is related to [4].

## Specifications table

**Table:** 

Subject area	Biology
More specific subject area	Pharmacokinetics, Toxicology
Type of data	Tables and Figures
How data was acquired	Human pharmacokinetic study, analytical chemistry (LC/MS/MS)
Data format	Analyzed and graphed
Experimental factors	Blood data and urine data analyzed before and after enzyme treatment
Experimental features	Human oral dosing of bisphenol A in soup
Data source location	N/A
Data accessibility	Within the data in brief article

## Value of the data

• Human blood concentrations of an endocrine active compound, bisphenol A.
• Individual data are provided for additional analysis.

## 1 Data

The age, body weight and body mass index of the study cohort is presented in Supplementary Table 1. Supplementary Tables 2–4 present supplemental multiple reaction monitoring (MRM) acquisition parameters for BPAS and BPAG, serum method validation results for d6-bisphenol A sulfate (BPAS^1^), and urine method validation results for d6-BPAS, respectively.

Commonly used blood collection devices were found in this study to introduce BPA into blood drawn through the apparatus (Supplementary Fig. 1). This result has been reported for other blood collection devices [6]. The items tested were: BD Nexiva catheter (BD 383516), Baxter Clearlink luer-activated valve (Baxter 2N8399), luer-lok access device (BD 364902), BD gold top vacutainer tubes (BD 368977), 10 ml saline flush syringe (BD 306546), 10 ml empty syringe (BD 309604), 12 G×1.25 in. blood collection needle (BD systems, 368607). Serum free of BPA was passed through apparatus composed of these materials.

The serum pharmacokinetic profiles of d6-BPA, d6-BPAG, d6-BPAS and d6-total BPA for each individual are shown in Supplementary Figs. 2, 3, 4, and 5, respectively.

Individual pharmacokinetic parameters for d6-BPA, d6-BPAS, d6-BPAG and d6-total BPA are presented in Supplementary Tables 5, 6, 7 and 8 respectively.

## 2 Experimental design, materials and methods

### 2.1 Materials and methods

All human subject research activities were conducted in accordance with protocols approved by the Pacific Northwest National Laboratory Institutional Review Board (IRB # 2014-14). The participation of the National Center for Toxicological Research was reviewed and approved by the FDA Research Involving Human Subjects Committee (RIHSC#10-119T) and determined not to constitute engagement in human subjects research because it was limited to analysis of anonymized samples.

### 2.2 Volunteer selection and demographics

Ten randomly selected healthy adult non-smoking (no nicotine product use) male volunteers were recruited for the study from the Salt Lake City, UT metropolitan area in 2014. Volunteers met criteria for normal organ function, no illicit drug use and normal oral mucosa.

### 2.3 Protocol

Volunteers were admitted to the clinical facility in the morning (Day 1), provided with breakfast one hour prior to ingesting d6-BPA then provided with 12 ounces of commercial tomato soup containing a 30 μg/kg b.w. dose of d6-BPA. Volunteers ingested the soup in their normal fashion. Venous blood samples were drawn immediately prior to ingestion of the soup, immediately after ingestion of the soup, and at regular intervals throughout the 24 h study period. All voided urine was collected at regular intervals for 13 h, then as volunteered for any additional voids, and again at the study conclusion, 24 h after ingestion.

### 2.4 Serum and urine collection

#### 2.4.1 Serum

Pre-study and terminal blood samples were drawn using a straight needle, single-use vacutainer holder, and BD gold-top serum collection tubes. All other blood samples were collected using components described in Teeguarden et al. [4]. Approximate blood sampling intervals were: pre-study, immediately after ingestion of the soup, then 10, 20, 30, 40, 60, and 90 min and 2, 3, 4, 5, 7, 9, 11, 13 and 24 h after ingestion of the soup. Actual sampling times for each volunteer are presented in graphical form in Supplementary Figs. 2–5.

#### 2.4.2 Urine

All expressed urine was collected in polypropylene urine hats (Kendall) over the course of the 24-hour study. Volume, weight and time were recorded. Urine sampling occurred immediately prior to ingestion of the soup, then at regular intervals corresponding to the timing of the blood samples (within logistical limits).

### 2.5 Serum and urine analysis of d6-BPA

Concentrations of total d6-BPA in serum and urine were quantified using LC/MS/MS as previously described [5], [4]. The method detection limit for total d6-BPA was 0.2 nM (0.05 ng/ml) in 10–100 µl aliquots of serum. Concentrations of d6-BPA in serum and urine were determined using the derivatization procedure described previously [2], [4]. For analysis of d6-BPA in 100 µl aliquots of serum, the limit of detection was 0.004 nM (0.001 ng/ml). Concentrations of BPA were quantified using LC/MS/MS as previously described [3], [1] and reported in [4]. Quality control measures were performed during every sample set.

### 2.6 Analysis of d6-BPA-glucuronide and d6-BPA-sulfate

Concentrations of d6-BPAG were quantified using LC/MS/MS and ^13^C_12_-BPAG as an internal standard [4] in a manner analogous to that previously described for BPAG [1]. The d6-BPAG (and d6-BPAS, see below) was not available as a standard so the same transitions used for the unlabeled BPAG (BPAS) were adjusted to the d6 variant. For analysis of d6-BPAG in 10 µl aliquots of serum, the limit of detection was 7 nM (3 ng/ml) and in 1 µl aliquots of urine, the limit of detection was 70 nM (30 ng/ml).

The LC method for d6-BPAS [4] was identical to that previously reported for BPAG [1] and the MRM transitions are listed in Supplementary Table 2. Limits of detection (*S*/*N*=3) and quantification for d6-BPAS in serum (10 µL aliquot) and urine (1 µL aliquot) were: Serum: LOQ, 0.4 ng/mL (1.3 nM) and LOD: 0.1 ng/mL (0.3 nM); Urine: LOQ, 10 ng/mL and LOD, 3 ng/mL.

### 2.7 Pharmacokinetic analysis

Plots of serum concentrations of total and d6-BPA at each time point following oral administration were analyzed using model-independent pharmacokinetic analysis (PK Solutions 2.0 software, Summit Research Services, Montrose, CO) as described [4].

## Footnotes

1 Abbreviations: The following terms were used throughout the manuscript in reference to forms of BPA: BPA, the unmetabolized, bioactive parent form; BPAG, BPA-glucuronide metabolite; BPAS, BPA-sulfate metabolite; Total BPA, the sum of BPA and all BPA conjugated metabolites. d6-, isotopically (deuterium) labeled forms; ^13^C_12_, ^13^C isotopically labeled forms. NCTR, National Center for Toxicological Research; bw, body weight.

Appendix A Supplementary data associated with this article can be found in the online version at doi:10.1016/j.dib.2015.03.002.
