# Supplementary material for: 24-hour human urine and serum profiles of bisphenol A following ingestion in soup: Individual pharmacokinetic data and emographics
Source: Data Brief. 2015 Mar 17;4:83–6. doi: 10.1016/j.dib.2015.03.002 (PMC4510366; doi:10.1016/j.dib.2015.03.002)
Supplement: Supplementary file 1 — Supplementary data [file mmc1.doc]

**Table 1: Volunteer Demographics**

| **Volunteer** | **Age (y)** | **Weight (kg)** | **Body Mass Index** |
| --- | --- | --- | --- |
| 002 | 23 | 68.9 | 20.3 |
| 005 | 29 | 69.5 | 21.8 |
| 006 | 21 | 83 | 26.1 |
| 008 | 35 | 85.4 | 26.6 |
| 011 | 30 | 91.8 | 27.2 |
| 014 | 23 | 85 | 23.1 |
| 015 | 25 | 76.4 | 21.4 |
| 016 | 29 | 77.7 | 24.5 |
| 017 | 45 | 111 | 35 |
| 018 | 25 | 64.1 | 19.1 |
|  |  |  |  |
| Average | 28.5 | 81.3 | 24.5 |
| Min | 21 | 64.1 | 19.1 |
| Max | 45 | 111 | 35 |

Table 2: MRM acquisition parameters for BPAS and BPAG

|  | **MRM** | **Dwell**  **(secs)** | **Cone (V)** | **Col. Energy** | **Delay (sec)** | **Compound** |
| --- | --- | --- | --- | --- | --- | --- |
| 1 | 306.9 - 212.0 | 0.07 | 40 | 30 | 0.005 | Native BPAS Confirm |
| 2 | 306.9 - 227.0 | 0.07 | 40 | 25 | 0.005 | Native BPAS Main |
| 3 | 312.9 - 215.0 | 0.07 | 40 | 30 | 0.005 | D6-BPAS Confirm |
| 4 | 312.9 - 233.0 | 0.07 | 40 | 25 | 0.005 | D6-BPAS Main |
| 5 | 318.9 - 224.0 | 0.07 | 40 | 30 | 0.005 | 13C-BPAS Confirm |
| 6 | 318.9 - 239.0 | 0.07 | 40 | 25 | 0.005 | 13C-BPAS Main |
| 7 | 402.9 - 113.1 | 0.07 | 30 | 20 | 0.005 | Native BPAG Confirm |
| 8 | 402.9 - 227.0 | 0.07 | 30 | 30 | 0.005 | Native BPAG Main |
| 9 | 408.9 - 113.1 | 0.07 | 30 | 20 | 0.005 | D6-BPAG Confirm |
| 10 | 408.9 - 233.0 | 0.07 | 30 | 30 | 0.005 | D6-BPAG Main |
| 11 | 414.9 - 113.1 | 0.07 | 30 | 20 | 0.005 | 13C-BPAG Confirm |
| 12 | 414.9 - 239.0 | 0.07 | 30 | 30 | 0.005 | 13C-BPAG Main |

**Table 3: Serum Method Validation Results for BPAS**

|  | **Spike Amount (ng/mL)** | | | | | |
| --- | --- | --- | --- | --- | --- | --- |
|  | **Avg ± SD (RSD), n=4** | | | | | |
|  | **2.5** | **5** | **10** | **25** | **50** | **100** |
| **Day 1** | 2.66 ± 0.12 (4%) | 4.83 ± 0.25 (5%) | 9.29 ± 0.31 (3%) | 22.7 ± 0.41 (2%) | 47.3 ± 1.82 (4%) | 93.5 ± 3.31 (4%) |
| **Day 2** | 2.40 ± 0.05 (2%) | 4.62 ± 0.16 (4%) | 9.74 ± 0.43 (4%) | 24.5 ± 0.47 (2%) | 47.6 ± 0.98 (2%) | 93.4 ± 4.39 (5%) |

Table 4: Urine Method Validation Results for BPAS

|  | **Spike Amount (ng/mL)** | | |
| --- | --- | --- | --- |
|  | **Avg ± SD (RSD), n=4** | | |
|  | **10** | **100** | **1000** |
| Day 1 | 10.88 ± 1.93 (18%) | 93.00 ± 2.71 (3%) | 985.47 ± 11.47 (1%) |
| Day 2 | 11.09 ± 0.74 (7%) | 102.7 ± 6.40 (6%) | 988.73 ± 75.75 (8%) |

Table 5. Individual Volunteer d6-BPA PK Parameters

| **Volunteer** | **T^1/2^  (h)** | | | **C_max_ (nM)** | **T_max_**  **(h)** | **AUC_0-∞_ (nmol×h x L^−1^)** |
| --- | --- | --- | --- | --- | --- | --- |
|  | **Elimination** | **Distribution** | **Absorption/Appearance** |  |  |  |
| **2** | 2.8 | 3.0 | 0.45 | 0.3 | 1.6 | 1.6 |
| **5** | 6.3 | 1.4 | 0.59 | 0.4 | 2.2 | 2.3 |
| **6** | 3.0 | 2.0 | 0.47 | 0.3 | 2.1 | 1.5 |
| **8** | 5.3 | 0.81 | 0.45 | 0.4 | 1.7 | 2.3 |
| **11** | 7.3 | 2.9 | 0.28 | 0.5 | 1.1 | 3.0 |
| **14** | 6.6 | 0.38 | 0.33 | 0.4 | 2.2 | 3.3 |
| **15** | 5.4 | 1.4 | 0.46 | 0.6 | 2.1 | 2.5 |
| **16** | 5.5 | 2.3 | 0.37 | 0.3 | 1.7 | 1.7 |
| **17** | 7.7 | 1.2 | 0.60 | 0.7 | 0.5 | 5.7 |
| **18** | 5.0 | 0.67 | 0.47 | 0.3 | 1.2 | 1.4 |

Table 6. Individual PK Parameters for d6-BPAS

| **Volunteer** | **T^1/2^ Appearance**  **(h)** | **C_max_ (nM)** | **T_max_**  **(h)** | **AUC_0-∞_ (nmol×h x L^−1^)** |
| --- | --- | --- | --- | --- |
| **2** | 0.41 | 14.3 | 2.1 | 106.8 |
| **5** | 0.32 | 13.3 | 1.2 | 80.2 |
| **6** | 0.45 | 17.7 | 1.6 | 74.9 |
| **8** | 0.55 | 13.1 | 2.2 | 54.9^a^ |
| **11** | 0.53 | 19.3 | 2.1 | 113.7 |
| **14** | 0.38 | 29.9 | 1.7 | 298.3 |
| **15** | 0.60 | 25.2 | 1.7 | 188.1 |
| **16** | 1.6 | 10.4 | 3.2 | 56.6 |
| **17** | 1.2 | 20.2 | 5.2 | 166.8 |
| **18** | 0.23 | 20.9 | 1.2 | 171.1 |

^a^The 24 hour zero BPAS concentration precluded calculation of an accurate AUC**_0-∞_** for volunteer 8. AUC_0-t_ calculated directly from the data was used. In all other volunteers, there was a minimal difference between AUC**_0-∞_** the AUC0-t calculated directly from the data.

Table 7. Individual PK Parameters for d6-BPAG

| **Volunteer** | **T^1/2^**  **Appearance**  **(h)** | **C_max_ (nM)** | **T_max_**  **(h)** | **AUC_0-∞_ (nmol×h x L^−1^)** |
| --- | --- | --- | --- | --- |
| **2** | 0.15 | 386 | 0.8 | 703.7 |
| **5** | 0.21 | 348 | 0.8 | 711.2 |
| **6** | 0.15 | 280 | 0.8 | 638.3 |
| **8** | 0.424 | 173 | 2.2 | 979.6 |
| **11** | 0.28 | 280 | 1.1 | 570.8 |
| **14** | 0.20 | 373 | 0.8 | 846.5 |
| **15** | 0.25 | 267 | 0.9 | 769.3 |
| **16** | 0.39 | 265 | 1.2 | 702.1 |
| **17** | 0.59 | 179 | 2.2 | 758.2 |
| **18** | 0.30 | 308 | 0.8 | 1214 |

Table 8. Individual PK Parameters for Total d6-BPA

| **Volunteer** | **T^1/2^ Appearance**  **(h)** | **C_max_ (nM)** | **T_max_**  **(h)** | **AUC_0-∞_ (nmol×h x L^−1^)** |
| --- | --- | --- | --- | --- |
| **2** | 0.26 | 492 | 0.8 | 1114 |
| **5** | 0.13 | 464 | 0.8 | 1201 |
| **6** | 0.23 | 364 | 0.8 | 1033 |
| **8** | 0.50 | 266 | 1.7 | 1044 |
| **11** | 0.29 | 383 | 0.8 | 962 |
| **14** | 0.27 | 474 | 0.8 | 1308 |
| **15** | 0.27 | 316 | 0.9 | 1041 |
| **16** | 0.45 | 326 | 1.2 | 1037 |
| **17** | 0.61 | 232 | 2.2 | 1338 |
| **18** | 0.26 | 353 | 0.8 | 1095 |


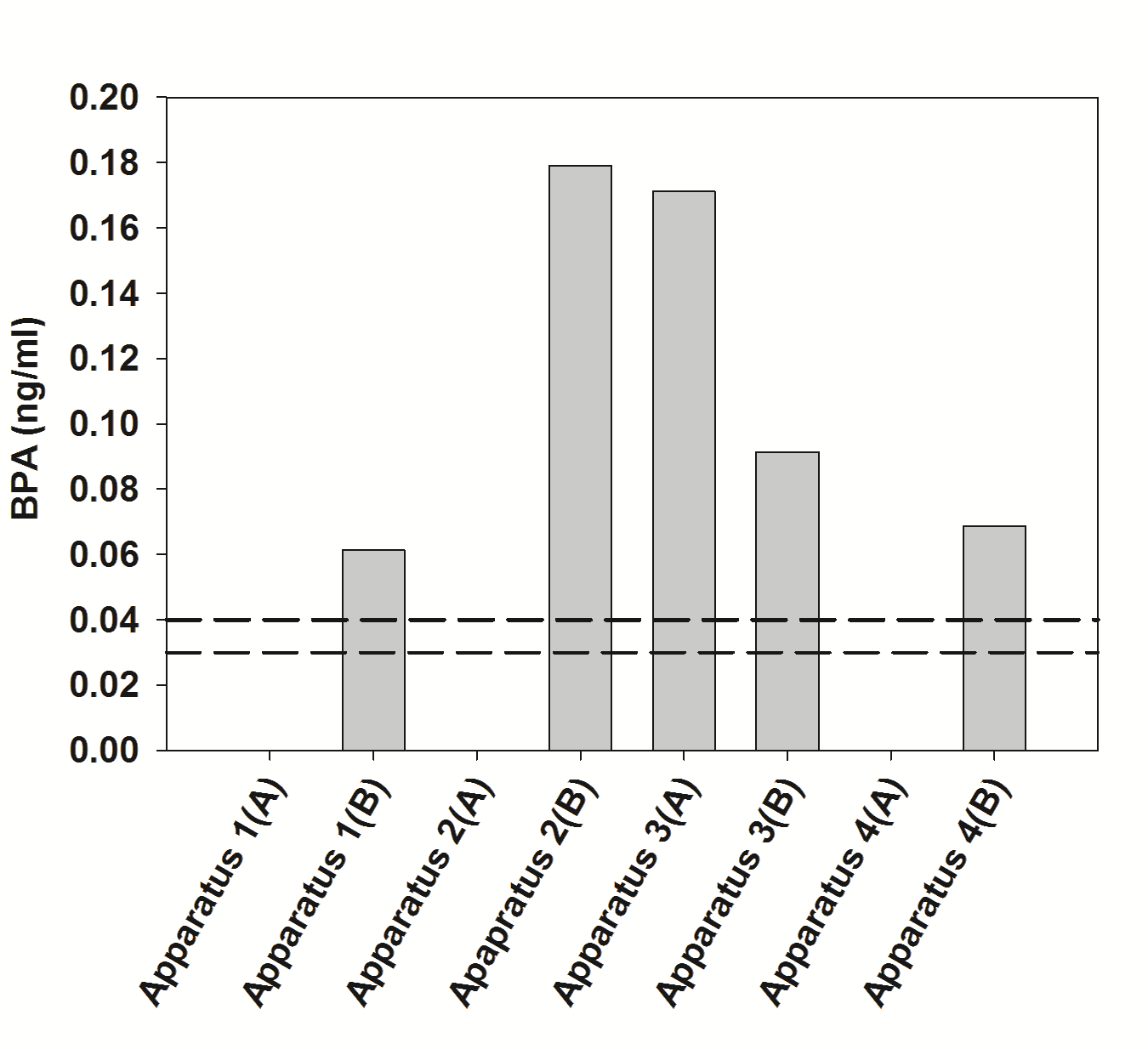


**Figure 1. Contamination of Blood Collection Devices by BPA**

BPA-free rat serum was drawn 2 times through 4 complete sets of blood collection materials. Some first draws (labeled A) were free of BPA, but all second draws (labeled B) had levels of BPA above the limits of blank (LOB), indicative of sample contamination during collection. The dashed lines represent the range of daily LOB for BPA (Churchwell et al., 2014; Teeguarden et al., 2015).

**Figure 2. Serum d6-BPA time-course for each volunteer**


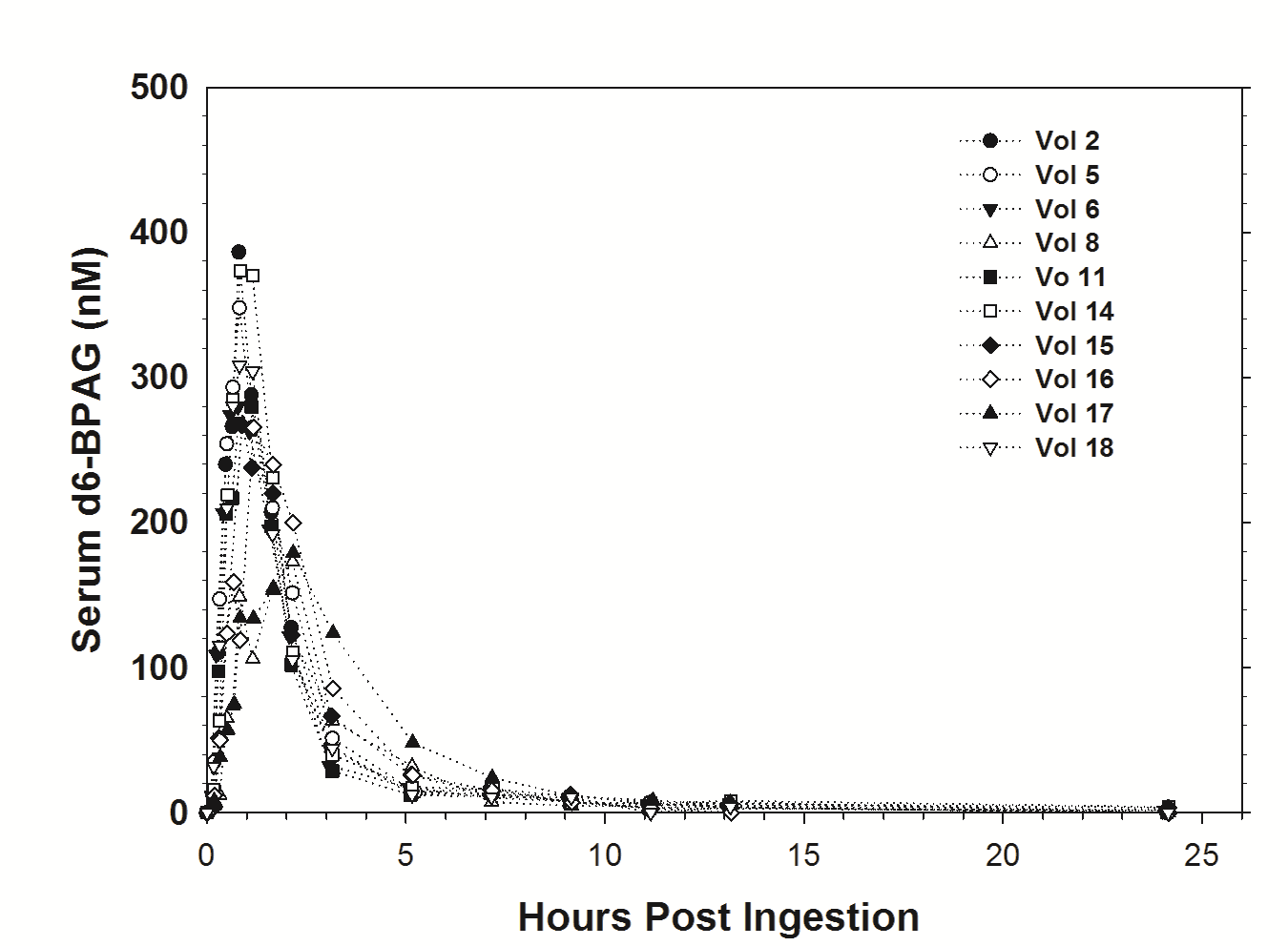


**Figure 3. Serum d6-BPAG time-course for each volunteer**


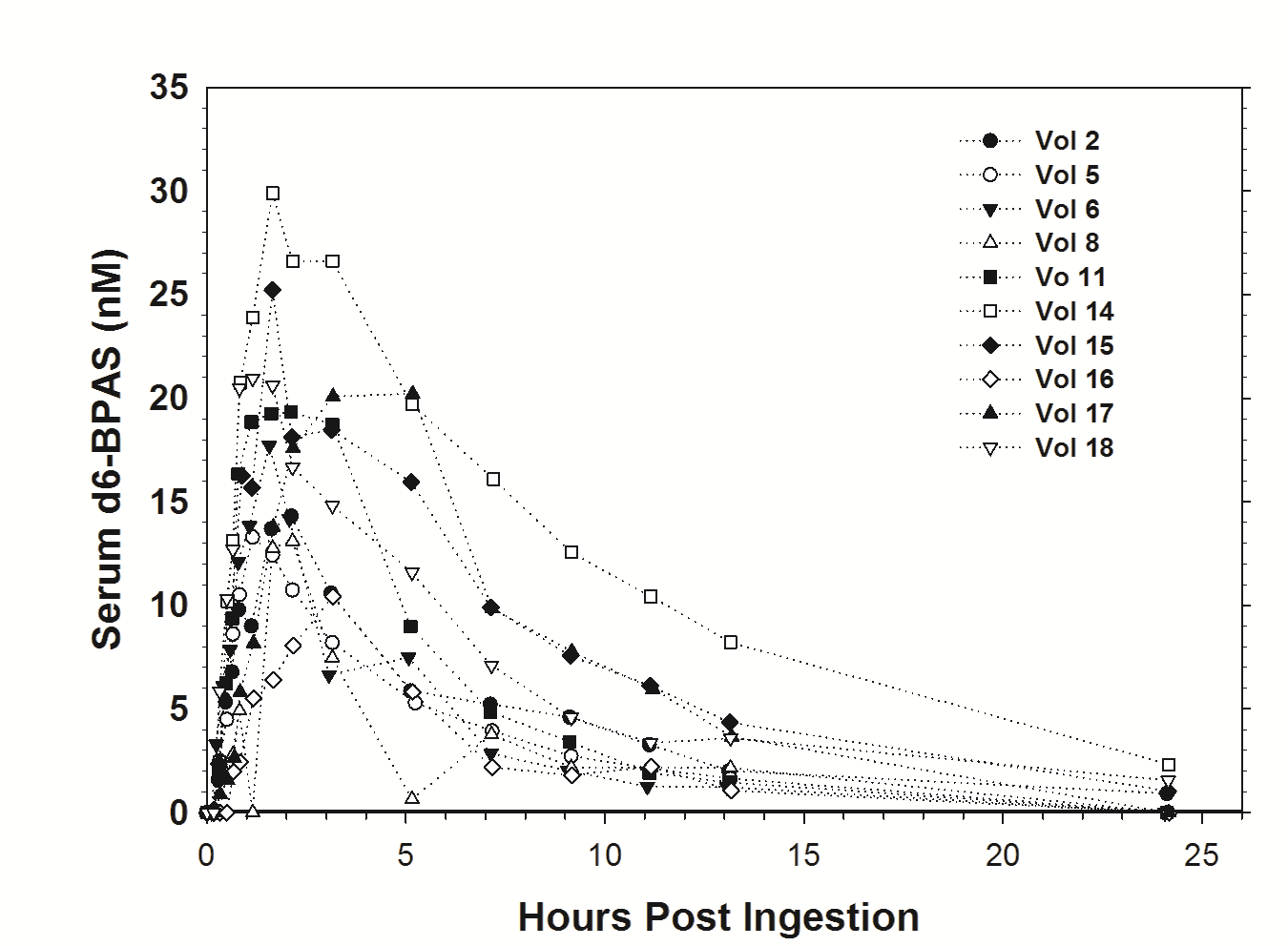


**Figure 4. Serum d6-BPAS time-course for each volunteer**


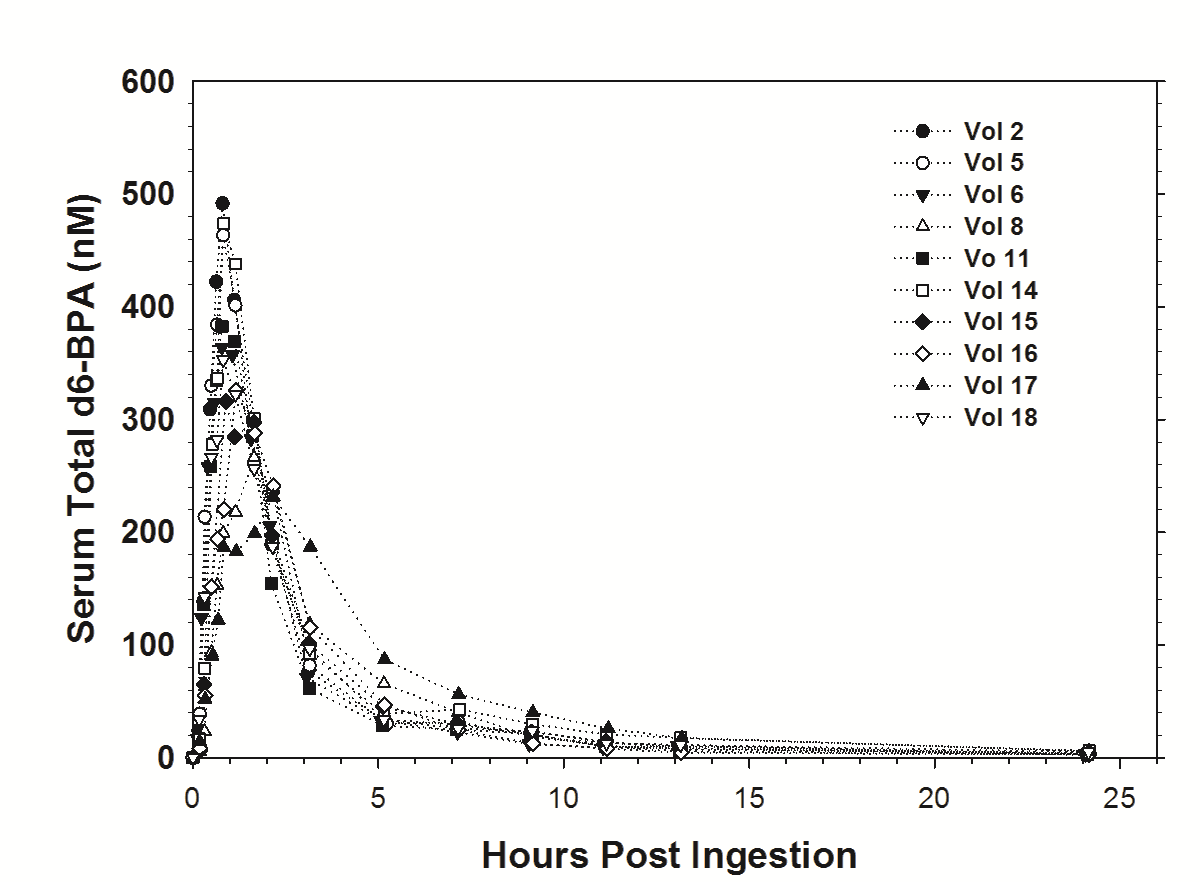


**Figure 5. Serum total d6-BPA time-course for each volunteer**
